# Evaluating crystallographic likelihood functions using numerical quadratures

**DOI:** 10.1107/S2059798320008372

**Published:** 2020-07-27

**Authors:** Petrus H. Zwart, Elliott D. Perryman

**Affiliations:** a Center for Advanced Mathematics in Energy Research Applications, Computational Research Division, Lawrence Berkeley National Laboratory, 1 Cyclotron Road, Berkeley, CA 94720, USA; b Molecular Biophysics and Integrative Bioimaging Division, Lawrence Berkeley National Laboratory, 1 Cyclotron Road, Berkeley, CA 94720, USA; c The University of Tennessee at Knoxville, Knoxville, TN 37916, USA

**Keywords:** maximum likelihood, refinement, numerical integration

## Abstract

Intensity-based likelihood functions in crystallographic applications have the potential to enhance the quality of structures derived from marginal diffraction data. Their usage, however, is complicated by the ability to efficiently compute these target functions. Here, a numerical quadrature is developed that allows the rapid evaluation of intensity-based likelihood functions in crystallographic applications. By using a sequence of change-of-variable transformations, including a nonlinear domain-compression operation, an accurate, robust and efficient quadrature is constructed. The approach is flexible and can incorporate different noise models with relative ease.

## Abstract 2

A quadrature is developed that allows the efficient evaluation of an intensity-based likelihood target function that includes experimental errors.

## 1. Introduction   

The estimation of model parameters from experimental observations plays a central role in the natural sciences, and the use of likelihood-based methods has been shown to yield robust estimates of ‘best guess’ values and their associated confidence intervals (Rossi, 2018 ▸). Maximum-likelihood estimation goes back to sporadic use in the 1800s by Gauss (Gauss, 1809 ▸, 1816 ▸, 1823 ▸) and Hagen (1867 ▸), and was further developed by Fisher (1915 ▸), Wilks (1938 ▸), and Neyman and Pearson (Neyman & Scott, 1948 ▸; Pearson, 1970 ▸). In the crystallo­graphic community, Beu *et al.* (1962 ▸) were the first to explicitly use maximum-likelihood estimation, applying it to lattice-parameter refinement in powder diffraction. In a late reaction to this work, Wilson (1980 ▸) states that ‘the use of maximum likelihood is unnecessary, and open to some objection’, and subsequently recasts the work of Beu *et al.* (1962 ▸) into a more familiar least-squares framework. It is important to note that least-squares estimation methods are equivalent to a likelihood formalism under the assumption of normality of the random variables. The use of maximum-likelihood-based methods using non-normal distributions in structural sciences took off after making significant impacts in the analysis of macromolecules. For these types of samples, structure solution and refinement problems were often problematic owing to very incomplete or low-quality starting models, making standard least-squares techniques underperform. In the 1980s and 1990s, likelihood-based methods became mainstream, culminating in the ability to routinely determine and refine structures that were previously thought to be problematic (Lunin & Urzhumtsev, 1984 ▸; Read, 1986 ▸; Bricogne & Gilmore, 1990 ▸; de La Fortelle & Bricogne, 1997 ▸; Pannu & Read, 1996 ▸; Murshudov *et al.*, 1997 ▸). A key ingredient to this success was the development of cross-validation techniques to reduce bias in the estimation of hyper-parameters that govern the behavior of the likelihood functions (Lunin & Skovoroda, 1995 ▸; Pannu & Read, 1996 ▸). At the beginning of the 21st century, Read and coworkers extended the likelihood formalism to molecular-replacement settings as well, resulting in a significant improvement in the ability to solve structures from marginal starting models (McCoy *et al.*, 2005 ▸; Storoni *et al.*, 2004 ▸; Read, 2001 ▸). The first use of approximate likelihood methods for the detection of heavy atoms from anomalous or derivative data originates from Terwilliger & Eisenberg (1983 ▸), who used an origin-removed Patterson correlation function for substructure solution. This approach was shown by Bricogne (1997 ▸) to be equivalent to a second-order approximation of a Rice-based likelihood function. A more recent development is the use of a more elaborate likelihood formalism in the location of substructures (Bunkóczi *et al.*, 2015 ▸), showing a dramatic improvement in the ability to locate heavy atoms. In density modification, the use of the likelihood formalism has significantly increased its radius of convergence (Terwilliger, 2000 ▸; Cowtan, 2000 ▸; Skubák *et al.*, 2010 ▸).

As the above examples illustrate, impressive progress has been made by the application of likelihood-based methods to a wide variety of crystallographic problems. In all of the described scenarios, key advances were made by deriving problem-specific likelihood functions and applying them to challenging structure-determination problems. In the majority of these cases, a thorough treatment of experimental errors has only a secondary role, resulting in approximations that work well in medium- or low-noise settings. The principal challenge in the handling of random noise in crystallographic likelihood functions is how to efficiently convolve Rice-like distribution functions modeling the distribution of a structure factor from an incomplete model with errors with the appropriate distribution that models the experimental noise. In this manuscript, we develop quadrature approaches to overcome these difficulties. We accomplish this by using a sequence of changes of variables that are amenable to straightforward numerical integration using standard methods. The approach derived has direct applications in model refinement and molecular replacement, while the general methodology can also be extended to other crystallographic scenarios. In the remainder of this paper, we will provide a general introduction to likelihood-based methods, provide a relevant background into numerical integration techniques, develop an adaptive quadrature approach, apply it to Rice-type likelihood functions and validate its results.

### 1.1. Maximum-likelihood formalism   

The estimation of model parameters **θ** given some data set 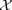 = {*x*_1_, …, *x_j_*, …, *x_N_*} via the likelihood formalism is performed in the following manner. Given the probability density function (PDF) *f*(*x_j_*|**θ**) of a single observation *x_j_* given a model parameter **θ**, the joint probability of the entire data set is, under the assumption of independence of the observations, equal to the product of the individual PDFs,
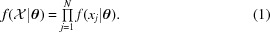
The probability of the data 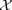 given the model parameters **θ** is known as the likelihood of the model parameters given the data:
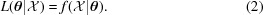
A natural choice for the *best estimate* of the model parameters is obtained by finding the **θ** that maximizes the likelihood function. This choice is called the maximum-likelihood estimate (MLE). The likelihood function itself 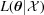 can be seen as a probability distribution, allowing one to obtain confidence-limit estimates on the MLE (Rossi, 2018 ▸). The determination of the MLE is typically performed by optimizing the log-likelihood:
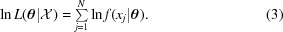
Often, the distribution needed for the likelihood function has to be obtained via a process known as marginalization. During this integration, a so-called nuisance parameter is integrated out,
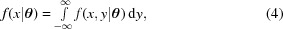
where, under the assumption of conditional independence, 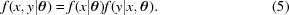
Depending on the mathematical form of the distributions involved, this marginalization can range from a trivial ana­lytical exercise to a numerically challenging problem. In likelihood functions in a crystallographic setting, this marginalization is required to take into account the effects of experimental noise, and its efficient calculation is the focus of this communication.

### 1.2. Motivation   

The most common likelihood function used in crystallo­graphic applications specifies the probability of the *true* structure-factor amplitude given the value of a calculated structure factor originating from a model with errors (Sim, 1959 ▸; Srinivasan & Parthasarathy, 1976 ▸; Luzzati, 1952 ▸; Woolfson, 1956 ▸; Lunin & Urzhumtsev, 1984 ▸):
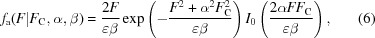


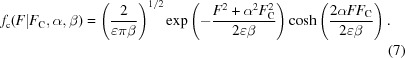

*f*_a_ and *f*_c_ are the distributions for acentric and centric reflections (the so-called Rice distribution), ∊ is a symmetry-enhancement factor, *F* is the true structure-factor amplitude and *F*_C_ is the current model structure-factor amplitude, while α and β are likelihood distribution parameters (Lunin & Urzhumtsev, 1984 ▸). For the refinement of atomic models given experimental data, the likelihood of the model-based structure-factor amplitudes given the experimental data is needed and can be obtained from a marginalization over the unknown, error-free structure-factor amplitude. Following Pannu & Read (1996 ▸) and assuming conditional independence between the distributions of the experimental intensity *I*_o_ and amplitude *F*, we obtain
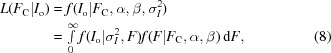
where *f*(*F*|*F*_C_, α, β) is given by expressions (6) or (7) and *f*(*I*_o_|σ_*I*_^2^, *F*) is equal to a normal distribution with mean *F* ^2^ and variance σ_*I*_^2^. This integral is equivalent to the MLI target function derived by Pannu & Read (1996 ▸). Because there is no fast-converging series approximation or simple closed-form analytical expression for this integral, various approaches have been developed, as excellently summarized by Read & McCoy (2016 ▸), including a method-of-moments-type approach to find reasonable analytical approximations to the intensity-based likelihood function.

In this work, we investigate the use of numerical integration methods to obtain high-quality approximations of integral (8) while also taking into account uncertainties in the estimated standard deviation. The approach outlined above, in which a Rice function is convoluted with a Gaussian distribution, essentially assumes that the standard deviation of the mean is known exactly. Given that both the standard deviation and the mean are derived from the same experimental data, this assumption is clearly suboptimal, especially when the redundancy of the data is low. In order to take into account possible errors in the observed standard deviation, we will use a *t*-distribution instead of a normal distribution, which arises as the distribution choice when the true standard deviation is approximated by an estimate from experimental data (Student, 1908 ▸). The aim of this work is to derive an efficient means of obtaining target functions that can provide an additional performance boost when working with very marginal data, such as those obtained from time-resolved serial crystallography or free-electron laser data, in which the experimental errors are typically larger than those obtained using standard rotation-based methods or have nonstandard error models (Brewster *et al.*, 2019 ▸). Furthermore, high-quality data sets are rarely resolution-limited by the diffraction geometry alone, indicating that many more marginal data are readily available that can potentially increase the quality of the final models if appropriate target functions are used. In the remainder of this manuscript, we develop and compare a number of numerical integration schemes aimed at rapidly evaluating intensity-based likelihood functions and their derivatives that take into account the presence of experimental errors, both in the mean intensity and in its associated standard deviation.

## 2. Methods   

In order to evaluate a variety of numerical integration schemes and approximation methods, the equations are first recast into a normalized structure-factor amplitudes *E* and normalized intensities *Z*_o_ framework, with the use of the σ_A_ formulation of the distributions involved, assuming a *P*1 space group, such that ∊ = 1 (Read, 1997 ▸). The joint probability distribution of the error-free structure-factor amplitude *E* and the experimental intensity *Z*_o_, given the calculated normalized structure factor *E*_C_, the model-quality parameter σ_A_, the estimated standard deviation of the observation σ_*Z*_ and the effective degrees of freedom ν, reads
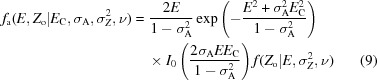
for acentric reflections and
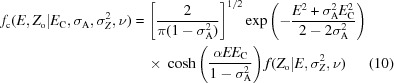
for centric reflections. When the distribution of the observed mean intensity *Z*_o_ is modeled by a *t*-distribution (Student, 1908 ▸) with a location parameter equal to *E* ^2^, we have
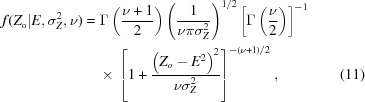
where ν is the effective degrees of freedom of the observation, which is related to the effective sample size *N*_eff_, 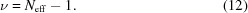
The effective sample size can be taken as the redundancy of an observed intensity, or can be estimated during data processing using the Welch–Satterthwaite equation (Welch, 1947 ▸) to take into account the weighting protocols implemented in data processing (Brewster *et al.*, 2019 ▸). The *t*-distribution arises as the distribution of choice given a sample mean and sample variance from a set of observations (Student, 1908 ▸). The use of a normal distribution essentially assumes no uncertainty in the variance σ_*Z*_^2^, but only in the observed mean *Z*_o_. The *t*-distribution is similar to a normal distribution, but has heavier tails and therefore will be expected to result in likelihood functions that are less punitive to larger deviations between observed and model intensities. When ν tends to infinity, the above distribution converges to a normal distribution, 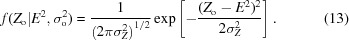
The above joint probability distributions need to be marginalized over *E* in 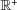 to obtain the distribution of interest:
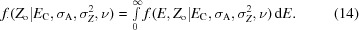



### 2.1. Variance inflation   

A common approach to avoid performing the integration specified above is to inflate the variance of the Rice function (1 − σ_A_^2^) by the variance of the ‘observed structure-factor amplitude’, yielding (1 − σ_A_^2^ + σ_E_^2^) (Green, 1979 ▸). This approach circumvents the need to perform an integration, but is suboptimal in a number of different ways. Because we do not observe amplitudes, we are required to estimate the amplitude and its variance from observed intensity data. A common way to perform the intensity-to-amplitude conversion is via a Bayesian estimate (French & Wilson, 1978 ▸) under the assumption of a uniform distribution of atoms throughout the unit cell. Although this so-called Wilson prior is used in most cases, a slightly different result can be obtained when using a constant, improper prior on the possible values of the structure-factor amplitudes on the positive half-line (Sivia & David, 1994 ▸). This results in an intensity-to-amplitude conversion that does not rely on the accurate estimation of the mean intensity, possibly complicated by the effects of pseudo-symmetry, diffraction anisotropy or twinning:
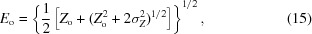


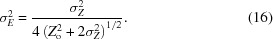
Further details are given in Appendix *E* [App appe]. While this procedure allows a straightforward intensity-to-amplitude conversion, even when intensities are negative, and can subsequently be used to inflate the variance of the Rice function, it is no substitute for the full integration. Given the simplicity of the variance-inflation approach and its wide usage in a number of crystallographic applications, we will use this approach as a benchmark, using conversion schemes based both on the Wilson prior (denoted French–Wilson) and on the outlined uniform, non-informative prior (denoted Sivia).

### 2.2. Approaches to numerical integration   

**Figure 1:**
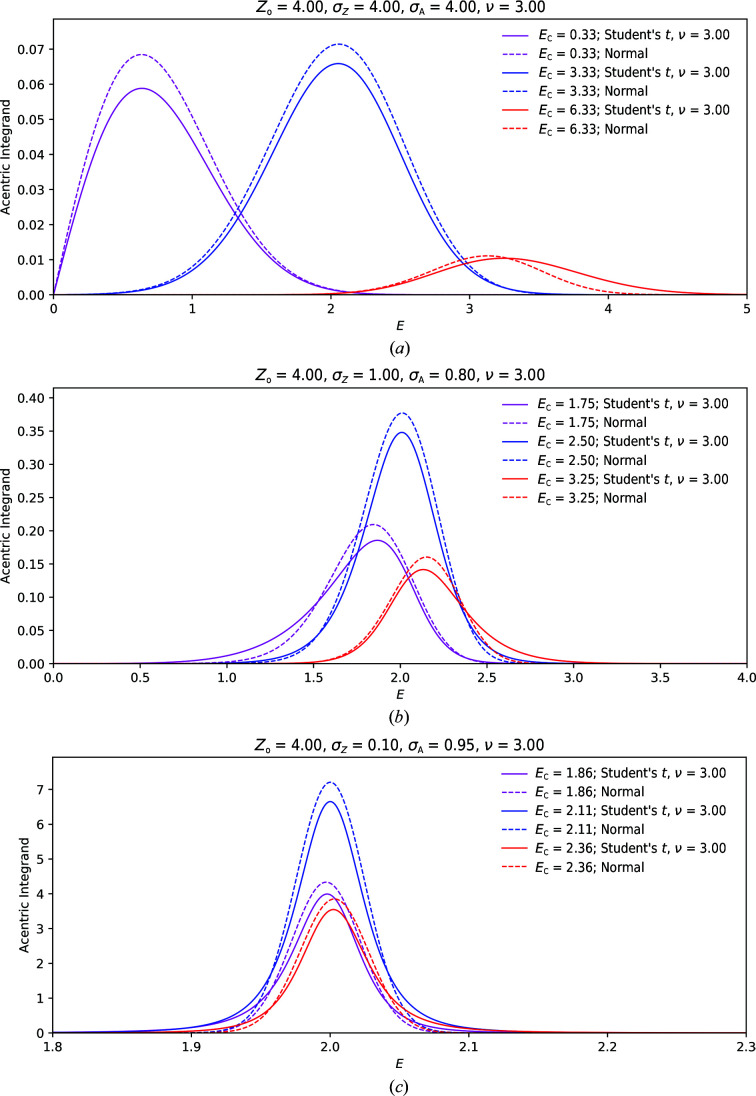
Integrand shapes for the acentric and centric distribution for different parameter settings show the variety of function shapes that occur when computing the marginal likelihood. When the experimental error is relatively large with respect to the intensity, high-mass areas of the function span a decent portion of the integration domain for *E* ≤ 6 (*a*). When the error on the experimental data is relatively small, the bulk of the integrand mass is concentrated in smaller areas (*b*, *c*). In the case of a *t*-distribution-based noise model, the tails of the distribution are lifted compared with the normal noise model. The variety of these shapes makes the uniform application of a standard quadrature or Laplace approximation inefficient and suboptimal.

Several conventional numerical integration approximations exist for improper integrals such as expression (8). Standard methods include trapezoidal-based methods with a truncated integration range, the use of Laplace’s method, Monte Carlo-based methods or approaches based on orthogonal polynomials (Davis & Rabinowitz, 1984 ▸). Whereas a straightforward use of a trapezoidal integration scheme is tempting, the shape of the integrand for certain combinations of distribution parameters will result in a fair chance of missing the bulk of the mass of the function unless a very fine sampling grid is used. When using the Laplace approximation, in which the integrand is approximated by an appropriately scaled and translated Gaussian function, the integrand can deviate significantly from a Gaussian, also resulting in a poor performance. These challenges are summarized in Fig. 1 ▸, where a number of typical integrand shapes are visualized for different parameter choices. A number of numerical integration and approximation methods will be outlined below, including a discussion of how *ground truth* is established as a basis for comparison. Here, we will limit ourself to the Laplace approximation owing to its simplicity and the trapezoidal rules because of their excellent convergence properties when applied to analytic functions on the real line and their close relation to classical Gauss quadratures (Trefethen & Weideman, 2014 ▸). The use of (quasi) Monte Carlo schemes will not be considered, since these methods are typically used as a ‘method of last resort’ for very high dimensional integrals (Cools, 2002 ▸).

### 2.3. Change of variables and the Laplace approximation   

Analytical and numerical integration is often greatly simplified by a change of variables of the integrand (Davis & Rabinowitz, 1984 ▸). The change-of-variable theorem relates the integral of some function *h*(*u*) under a change of variables *u* = ψ(*x*),
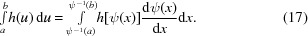
The modified shape of the integrand by a change of variables makes the use of the so-called Laplace approximation appealing. In a Laplace approximation, the integrand is approximated by a scaled squared exponential function with a suitably chosen mean and length scale (Peng, 2018 ▸). The Laplace approximation can be derived from truncated Taylor expansion of the logarithm of the integrand:
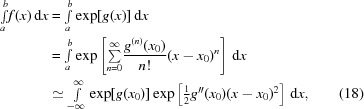
where *g*(*x*) = ln[*f*(*x*)] and *x*_0_ is the location of the maximum of *f*(*x*), implying that *g*′′(*x*_0_) = 0. Note that in the last step in equation (18) the assumption is made that *f*(*x*) goes to 0 when not near *x*_0_ quickly enough that integrating over [*a*, *b*] yields the same results as integrating over 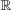. Although this approximation does not work for all possible choices of *g*(*x*), it has proven to be a successful tool in marginalizing distributions in Bayesian analysis (Kass & Steffey, 1989 ▸) and crystallographic applications (Murshudov *et al.*, 2011 ▸).

The above expression thus yields
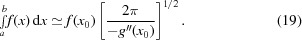
The effectiveness of this approximation hinges on the location of *x*_0_ (it should be contained within the original integration domain), the magnitude of *g*′′(*x*_0_) and how rapidly higher-order derivatives of *g*(*x*) vanish around *x*_0_. The change-of-variable strategy outlined above can aid in increasing the performance of approximation to expression (8).

### 2.4. Quadrature methods   

Even though the change-of-variables approach combined with the Laplace approximation has the potential to yield accurate integral approximations, obtaining reasonable estimates of the derivative of the log-likelihood, as needed for difference maps or for first or higher-order optimization methods, seems less straightforward using the Laplace approach. The difficulty arises from the need to obtain the derivative of the location of the maximum of integrand, as this value is a function of the variables for which derivatives are computed. In addition, the introduction of *t*-based noise models introduces heavy tails in the distribution for which Gaussian approximations can have a poor performance. For this reason, the use of a quadrature approach is of interest. In a numerical integration with a quadrature, the integral of interest is approximated by a weighted sum of selected function values. The Laplace approximation outlined above can thus be seen as a one-point quadrature, where the location of the function value is located at the maximum of the integrand, and the associated weight is derived from a local Gaussian fit to the integrand. An expanded quadrature approach provides an easy way to increase the precision of the integral by increasing the number of sampling points, but also circumvents issues with computing derivatives of the location of the maximum of the integrand that are encountered when using the Laplace approximation. Quadrature approaches have, however, been assumed to need a large number of terms to obtain sufficient precision (Read & McCoy, 2016 ▸), possibly making them an unattractive target for practical crystallographic applications. In order to circumvent or at least ameliorate these issues, we design quadratures that combine the benefits of a Laplace approximation and basic numerical quadratures (Appendix *A* [App appa]).

**Figure 2:**
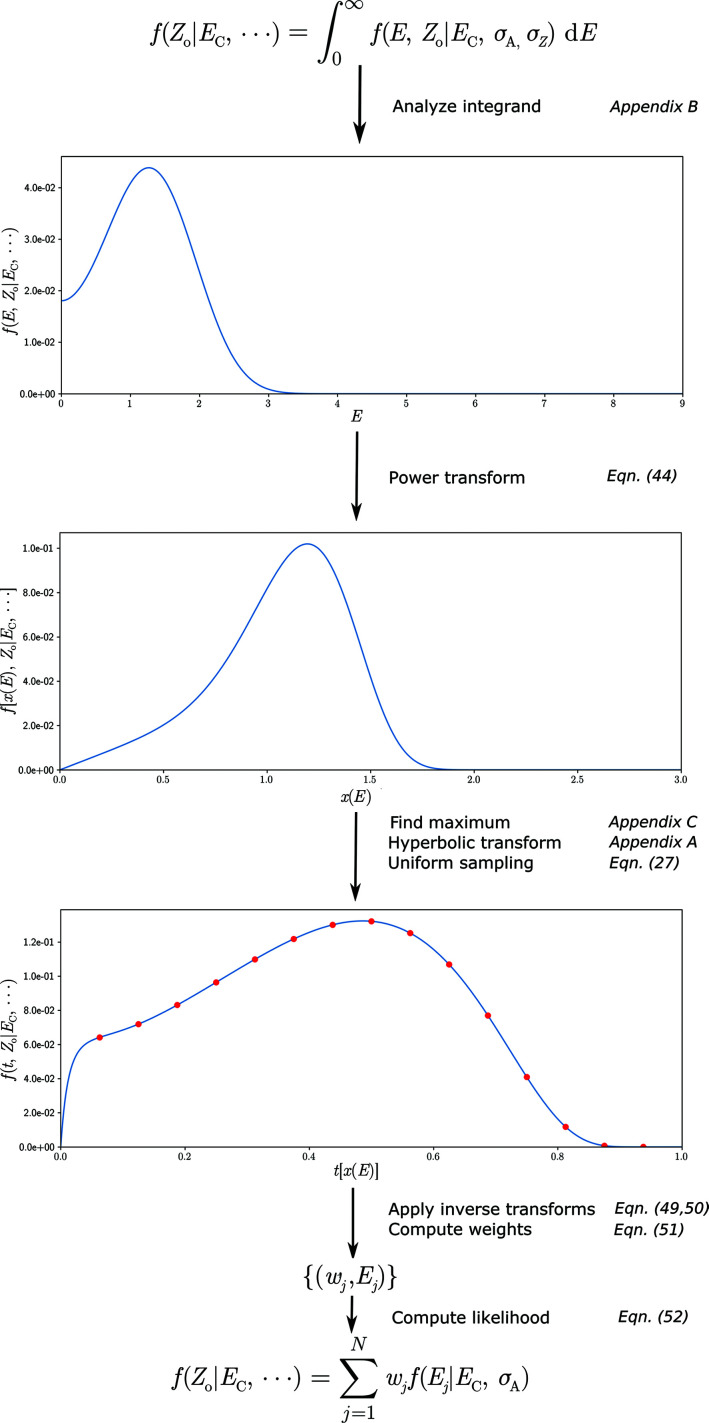
The numerical integration procedure developed is depicted as a sequence of steps. The general idea is to use a sequence of variable transformations that result in a smooth function on [0, 1] which can be easily integrated via a trapezoidal integration scheme. Once quadrature points have been established, the integration can be written as a sum of weighted Rice functions. See the main text for details.

A high-level overview of our integration approach is depicted in Fig. 2 ▸. By combining a power transform followed by a hyperbolic transform of the integrand, we transform the integration domain from [0, ∞] onto [0, 1]. While the first power transform (Appendix *C* [App appc]) allows the integrand to have more Gaussian-like character, the second change-of-variable operation nonlinearly compresses low-mass regions onto relatively small line segments, while approximately linearly transforming high-mass areas of the integrand to the middle of the new integration domain (Appendix *A* [App appa]). This double-transformed function can subsequently be integrated using an equidistant trapezoidal integration scheme. The second change-of-variable operation requires, just like the Laplace approximation, knowledge of the maximum of the power-transformed integrand, which can be obtained using standard optimization methods (Appendices *B* [App appb] and *C* [App appc]). In a final step, the resulting quadrature expressed on the domain of the doubly transformed variable can be rewritten in the original variables by applying inverse transforms. A subsequent further simplification allows us to recast the whole integration as a sum of weighted Rice functions, where the effects of noisy observations and other errors are *hidden* in the sampling of *E* on 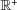 and the associated weights (Appendix *D* [App appd]),
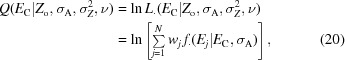
where *E_j_* are the quadrature sampling points and *w_j_* are the associated weights. The sampling points and weights are dependent on *E*_C_, *Z*_o_, σ_A_, σ_*Z*_ and ν. The quadrature sampling used can either be an *N*-point power-transformed hyperbolic quadrature or a single-point quadrature on the basis of a (power-transformed) Laplace approximation. Further details are given in Appendices *A*–*D* [App appa] [App appb] [App appc] [App appd].

### 2.5. Derivatives   

The practical use of a likelihood-based target function requires the calculation of its derivatives so that it can be used in gradient-based optimization methods. From expression (20), derivatives with respect to *Y* ∈ {*E*_C_, σ_A_, ν} can be obtained as follows:
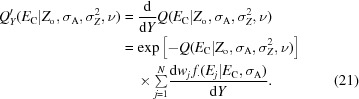
The derivatives of the Rice components *f*_·_(*E*_*j*_|*E*_C_, σ_A_) with respect to *E*_C_ are listed in Appendix *B* [App appb].

## 3. Results and discussion   

The first step in evaluating the proposed integration methods is to establish the ground truth of the integral that we wish to approximate. To this end, an equispaced, non-power-transformed trapezoidal quadrature was constructed integrating the function from *E* = 0 to *E* = 6 using 50 000 sampling points using all combinations of distribution parameters, as listed in Table 1 ▸, under the assumption of Gaussian errors on the intensities. Comparing the results of this integration with those obtained using a hyperbolic quadrature with 1500 points indicates that these two integration methods give similar results. We therefore take the ground truth as the results obtained with a hyperbolic quadrature using 1500 or more sample points. For both the acentric and the centric distributions, setting the power-transform variable γ to 2 provides good results, as shown in Tables 2 ▸ and 3 ▸, where the mean and standard deviation of the relative error in the log integrand are reported (as percentages). A number of different approximation schemes were tested, comparing results using the mean relative error in the log integrand. Because the variance-inflation approximation does not actually perform a marginalization, but performs a more *ad hoc* correction to incorporate low-fidelity measurements, its relative error against the log-likelihood is not a fair measure of its performance, nor does it provide insights into its strengths and drawbacks. Instead, we will compare the gradients of the log-likelihood target function with respect to *E*_C_ for all approximations, as this measure is independent of the different normalizations that arise when computing the full integral as compared with the variance-inflation approaches. Furthermore, given that the gradients of the log-likelihood function form are the Fourier coefficients of the 3D difference or gradient maps used to complete or rebuild structures, comparing the gradients of various approximations with those obtained from the full likelihood function can provide valuable insights into the strengths of different approximations. The use of gradients is of course predicated on being able to estimate the value of σ_A_, which in this case can be performed using a simple line search in fixed resolution shells. Details of these tests and their results can be found below.

### 3.1. Comparing integration methods   

**Figure 3:**
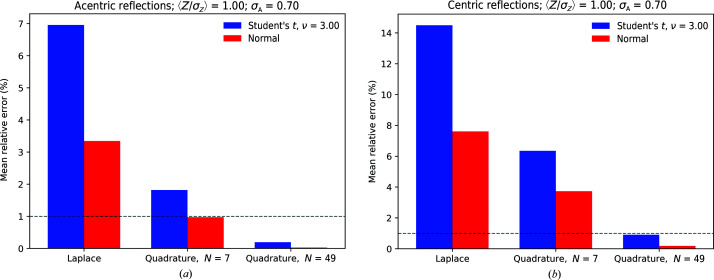
The relative mean error of the likelihood functions using a Laplace approximation and quadrature-based methods for normal and *t*-based noise models, for acentric (*a*) and centric reflections (*b*). The dotted horizontal line is set at 1% as a visual reference.

A comparison of the integration results using a number of different approximations are visualized in Fig. 3 ▸ for data sets generated according to the procedure outlined in Appendix *F* [App appf]. For the results shown the value of σ_A_ was set to 0.70, and a fixed error ratio was chosen such that 〈*Z*/σ_*Z*_〉 = 1.0. The redundancy was set to 4, resulting in ν = 3. For the *Z*_o_, *E*_C_ pairs, a likelihood function computed using a 1500-point hyperbolic quadrature was treated as the ground truth both for a *t*-distribution and an error model assuming a normal distribution. These values were compared with the Laplace approximation (a one-point quadrature) and seven-point and 49-point quadratures for both error models. While the Laplace approximation behaves relative well for the normal error model, it fails to deal properly with the elevated tails of the *t*-distribution, and better results are obtained using a quadrature. Satisfactory results are obtained using quadratures composed of seven or more sampling points. General heuristics can in principle be developed to tailor the specific accuracy of the quadrature on the basis of the hyperparameter of the error model. As expected, *t*-distributions with low ν values require a larger quadrature to get to a comparable error compared with those originating from a normal distribution owing to the presence of heavier tails.

### 3.2. Comparing likelihood functions   

**Figure 4:**
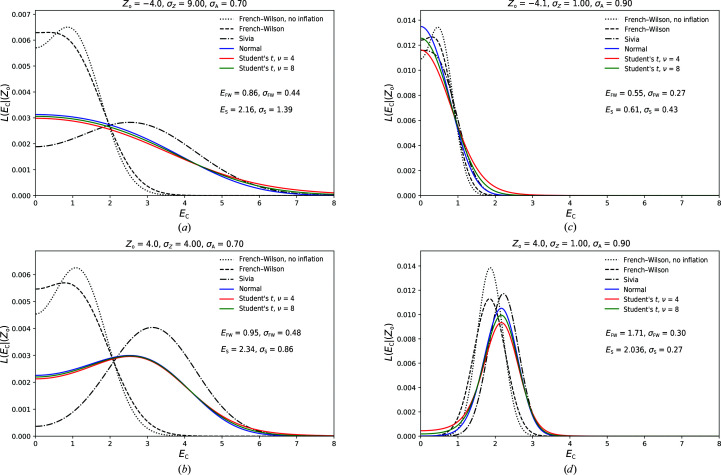
The shape of normalized likelihood functions under a number of different approximations for different input parameters indicates that the use of a point estimate for negative intensities or those with high noise values results in significant deviations from the ideal likelihood function. The difference between a *t*-based noise model and a normal noise model is small, but significantly affects the tail behavior of the likelihood function. Amplitude and standard deviation estimates for both the French–Wilson and Sivia approaches are given in the figure.

**Figure 5:**
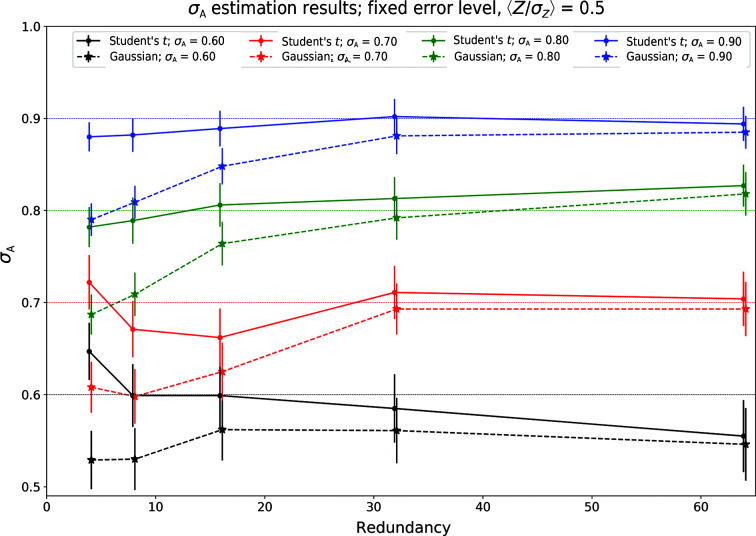
The behavior of a likelihood-based σ_A_-estimation procedure when data with a *t*-based noise model are treated with a likelihood-based approach using normal noise: a negative bias is introduced in the estimate of σ_A_ at low redundancies.

**Figure 6:**
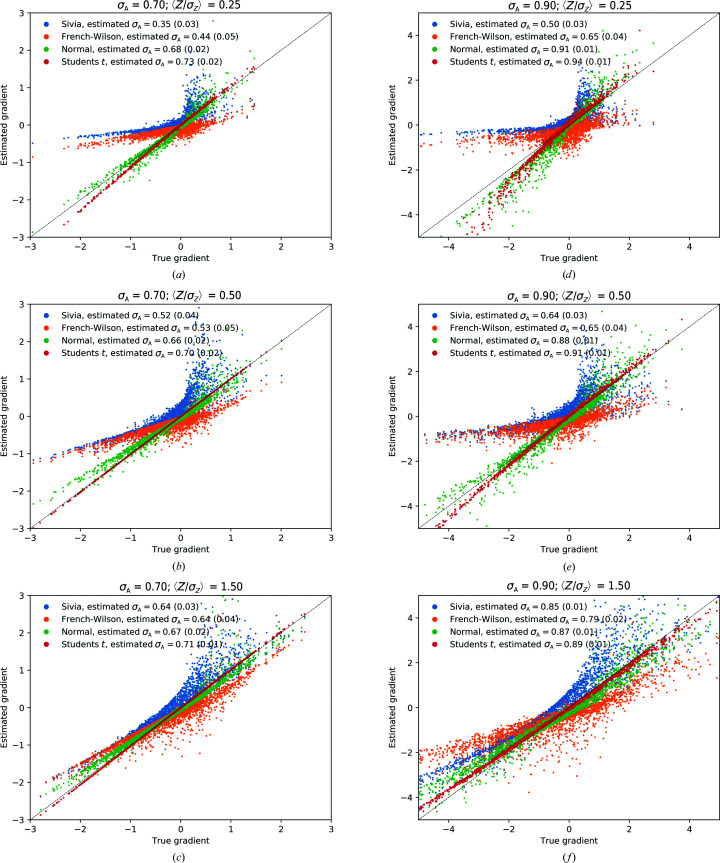
A comparison of gradients computed using different approximation schemes for *t*-based noise with ν = 3. (*a*)–(*c*) and (*d*)–(*f*) depict the behavior of the gradient approximations with a decreasing noise level. Gradients were computed using a maximum-likelihood estimate of σ_A_ using their corresponding approximations. Both the normal model and the *t*-based model clearly outperform the French–Wilson and the Sivia approaches, while a marginal improvement over the normal noise model is obseved when the *t*-based model is used.

In order to obtain a better intuition of the behaviors of the target functions, we directly plot them for a few input parameters. Fig. 4 ▸ depicts the likelihood function *L*(*E*_C_|*Z*_o_) for acentric reflections using just the French–Wilson protocol to estimate the amplitude while not inflating the variance and using the variance-inflation method with both the French–Wilson and the Sivia approaches, as well as the full likelihood functions using both a Gaussian error model and a *t*-distribution variant. All functions shown have been numerically normalized over 0 ≤ *E*_C_ ≤ 12. When comparing the curves for weak and negative intensities, there is a remarkably large difference between tech­niques that use an estimate of *E*_o_ on the basis of a non-informative prior (French–Wilson & Sivia) versus those derived by the full integration (Figs. 4 ▸ *a*, 4 ▸ *b* and 4 ▸ *c*). In the case of an observation with lower associated standard deviation, the differences between the approximations are smaller. The differences between a normal error model and a *t*-distribution manifest themselves in the tail behavior of the likelihood-function approximations, while the locations of the maxima seem relatively unchanged (Fig. 4 ▸ *d*). The practical effects of the mismatch between an assumed normal error model and the *t*-type error models become apparent in the estimation of σ_A_ on the basis of the corresponding likelihood approximation. A synthetic data set with errors was constructed using the protocol outlined in Appendix *F* [App appf]. The errors were chosen using a fixed error level such that the expected 〈*Z*_o_/σ_*Z*_〉 was 0.5 when ν → ∞ (see Appendix *F* [App appf]). The resulting *Z*_o_, σ_*Z*_ and *E*_C_ values were used to determine σ_A_ via a golden section-driven likelihood-maximization procedure (Kiefer, 1953 ▸). The resulting estimates of σ_A_ and their associated estimated standard deviation for different redundancy values (ν + 1) are shown in Fig. 5 ▸. While for large values of ν the estimated values of σ_A_ are equivalent for both error models, at lower redundancy values the normal error model systematically underestimates σ_A_. When the French–Wilson protocol is used, the resulting σ_A_ estimates are underestimated even more (Fig. 6 ▸).

### 3.3. Comparing log-likelihood gradients   

Additional insights into the behavior of the likelihood-function approximation can be obtained by directly comparing its gradients for a selected set of parameter combinations. Numerical tests indicate that gradients computed using a 1500-point hyperbolic quadrature of the power-transformed function (with γ set to 2 for both the acentric and centric distribution) are indistinguishable from finite-difference gradients computed with a 50 000-point trapezoidal approach. In order to investigate the quality of the various approximations under common refinement scenarios, we construct a synthetic data set using random sampling methods as outlined in Appendix *F* [App appf]. A redundancy of 4 (ν = 3) was used in these tests. Gradients were computed using a 49-point quadrature, using a value of σ_A_ estimated from the corresponding approximation to the likelihood function. The results of these comparisons are shown in Fig. 6 ▸ and summarized in Tables 4 ▸ and 5 ▸. The quality of the gradients is gauged by a correlation coefficient to the true value. The results indicate that for data for which 〈*Z*_0_/σ_*Z*_〉 is large, all gradient-calculation methods converge to those obtained using the full intensity-based likelihood function with experimental errors and a Student’s *t* noise model, but that for high and intermediate noise levels the variance-inflation method significantly underperforms. While differences between normal and *t*-style noise models seem small on the basis of the correlation coefficients, significant deviations are seen in individual reflections under high-noise and low-redundancy settings. These aberrant gradients can potentially negatively influence the quality of gradient maps for structure completion.

## 4. Conclusions   

Numerical procedures for the efficient determination of intensity-based likelihood functions and their gradients are developed and compared. Whereas the Laplace approximation behaves reasonably well for the estimation of the likelihood function itself under a normal noise model, our results show that the both the likelihood and its associated gradients can be significantly improved upon by using a numerical quadrature. Given that the derivative of the log-likelihood function is the key ingredient in gradient-based refinement methods and is used to compute difference maps for structure completion, the proposed approach could improve the convergence of existing refinement and model-building methods. Although it is unclear what the optimal quadrature order or noise model should be in a practical case, our results suggest that it is likely to be below 15 sampling points for normal noise and below 49 for *t*-type errors. Algorithmically, the most costly operation is the iterative procedure for finding the maximum of the integrand. The proposed Newton-based method typically converges well within 50 function evaluations, even in the absence of a predetermined good starting point for the line search. The construction of the hyperbolic quadrature does not require any iterative optimization, nor does the subsequent calculation of the associated gradient and function values. Given the large additional overhead in refinement or other maximum-likelihood applications in crystallography, the use of the presented methodology to compute target functions is likely to have only have a minimal impact on the total run time of the workflow, while providing a rapidly converging approximation to a full intensity-based likelihood that takes experimental errors in both the estimate of the mean intensity and its variance into account. Although only a full integration into a crystallographic software package can determine the situations under which a practical benefit can be obtained from using the outlined approach, the tests here indicate that significant improvements are possible. Furthermore, the ease with which the proposed quadrature method can be adapted to a different of choice of error model is a large benefit over existing approximation methods, making it for instance possible to use experiment-specific noise models in refinement and phasing targets (Sharma *et al.*, 2017 ▸).

## Figures and Tables

**Table 1:** Parameter bounds for comparing integration methods

Parameter	Start	End	Sampling points
*E*_C_	0.1	6.0	20
σ_A_	0.0	0.95	10
*Z*_o_	−5.0	50.0	20
*Z*/σ_*Z*_	0.5	10.0	20

**Table 2:** Integration results: acentric distribution The mean error and standard deviation of the relative log-likelihood over the full parameter range are reported as percentages.

Method	γ = 1	γ = 2	γ = 3
Laplace approximation	−0.142/0.874	0.294/0.971	0.485/1.135
Quadrature (*N* = 3)	0.191/0.778	0.152/0.831	0.281/1.058
Quadrature (*N* = 5)	0.130/0.377	0.126/0.481	0.196/0.627
Quadrature (*N* = 7)	0.085/0.218	0.074/0.309	0.116/0.428

**Table 3:** Integration results: centric distribution The mean error and standard deviation of the relative log-likelihood over the full parameter range are reported as percentages. Quadrature results for γ = 1 are absent because the function is not guaranteed to be zero at the origin as required by the hyperbolic quadrature scheme.

Method	γ = 1	γ = 2	γ = 3
Laplace approximation	−1.766/8.170	0.357/1.729	0.738/1.841
Quadrature (*N* = 3)	—	0.300/1.617	0.725/1.850
Quadrature (*N* = 5)	—	0.391/0.990	0.438/1.183
Quadrature (*N* = 7)	—	0.269/0.750	0.311/0.943

**Table 4:** Comparing likelihood gradients for simulated data by computing correlations of gradients computed using a 1500-point quadrature with the correct σ_A_ value (0.70) and those obtained using four different approximation methods, as outlined in the main text, and the maximum-likelihood estimate of σ_A_ given the approximation of the likelihood function The reported entries are estimated values of σ_A_ and the gradient correlation.

Method	〈*Z*/σ_*Z*_〉 = 0.25	〈*Z*/σ_*Z*_〉 = 0.5	〈*Z*/σ_*Z*_〉 = 1.5
Sivia	0.35/68.8%	0.52/82.1%	0.64/93.5%
French–Wilson	0.44/80.5%	0.53/87.7%	0.64/94.3%
Normal	0.68/96.9%	0.66/97.4%	0.67/97.9%
Student’s *t*	0.73/100%	0.70/100%	0.71/100%

**Table 5:** Comparing likelihood gradients for simulated data by computing correlations of gradients computed using a 1500-point quadrature with the correct σ_A_ value (0.90) and those obtained using four different approximation methods, as outlined in the main text, and the maximum-likelihood estimate of σ_A_ given the approximation of the likelihood function The reported entries are estimated values of σ_A_ and the gradient correlation.

Method	〈*Z*/σ_*Z*_〉 = 0.25	〈*Z*/σ_*Z*_〉 = 0.5	〈*Z*/σ_*Z*_〉 = 1.5
Sivia	0.50/63.1%	0.64/73.6%	0.85/93.2%
French–Wilson	0.64/60.3%	0.65/74.9%	0.79/88.4%
Normal	0.91/97.0%	0.88/96.9%	0.87/97.6%
Student’s *t*	0.94/98.7%	0.91/99.9%	0.89/99.9%

## Appendix A A hyperbolic quadrature

Given a function *g*(*x*), with *x* ≥ 0 and *g*(*x*) ≥ 0, we seek to compute its integral over the positive half-line:

Set

Define the supremum of *g*(*x*) by *x*_0_ such that *h*′(*x*_0_) = 0. For the class of functions we are interested in, *g*(0) is equal to 0, for instance owing to the power transform outlined in the main text, and  is 0 as well. Define the following change of variables on the basis of a shifted and rescaled logistic function,

where *k* is a positive contant. Note that *t*(*x* = 0) = 0 and  = 1. The inverse function is

and has a derivative with respect to *t* equal to

The value *x*_0_ determines the approximate ‘inflection’ point of the hyperbolic compression scheme and the constant *k* controls the slope around the midpoint. An *N*-point quadrature can now be constructed by uniformly sampling *t* between 0 and 1,

for 1 ≤ *j* ≤ *N*. Given that both *g*(0) and  are zero, the integral *G* can now be computed via a trapezoidal integration rule,

If *k* is chosen to be

then the above quadrature for *N* = 1 yields the Laplace approximation when *x*_0_|*h*′′(*x*_0_)|^1/2^ is large, as |*x*_0_ − *x*(1/2)| goes to zero. If a hyperbolic quadrature is constructed on a distribution of power-transformed variables, these derived weights can be multiplied by the Jacobian of that transformation, so that the final numerical evaluation can be carried out in the original set of variables.

## Appendix B Distributions and derivatives

### b1. Rice functions, acentrics

The logarithms of the acentric Rice distribution and its derivatives with respect to *E* and *E*_C_ are given below.

### b2. Rice functions, centrics

The logarithms of the centric Rice distribution and its derivatives with respect to *E* and *E*_C_ are given below.

### b3. Student’s *t*-distribution

The logarithm of the *t*-distribution as specified in the main text and its derivatives with respect to *E* are given below.

When ν is large, the *t*-distribution can be approximated with a normal distribution:

## Appendix C Finding *x*_0_

As outlined in the main text, numerical integration via the hyperbolic quadrature is greatly assisted by the change of variables

with Jacobian (see expression 17)

The location of the maximum value of the integrand, *x*_0_, is found using a straightforward application of the Newton root-finding algorithm using the first and second derivatives that are outlined below. Set

and

Using the shorthand *h_t_*(*E*, ⋯) to indicate either of these functions, we first apply a power transform (expression 44), as outlined above. Because we need to locate the maximum of this function, we need the derivatives with respect to *x* as well. The resulting function and its first and second derivatives with respect to *x* after the change-of-variable operation are given by

The analytic forms for *h_t_*(⋯), *h*′_*t*_(⋯) and *h*′′_*t*_(⋯) are given in Appendix *B* [App appb]. Decent starting values for the Newton search can be found by performing a single Newton-based update on a set of (say) 15 equispaced values of *x* sampled between 0 and *x* = 6^1/γ^. The integrand-weighted mean of the resulting updated sampling points typically refines within ten iterations to the supremum.

## Appendix D Likelihood synthesis

Using the above approaches, the full likelihood function can be expressed as a sum of weighted Rice functions (expressions 30 and 34), where *E* is sampled on the basis of a quadrature derived from a power-transformed variable *E* = *x* ^γ^ using the hyperbolic sampling scheme outlined above. Taking into account the combination of the power transform and the hyperbolic quadrature, the sampling nodes of the quadrature are equal to

where *t_j_*, *x*_0_ and *k* are defined and computed as outlined in Appendices *A* [App appa] and *C* [App appc] and 1 ≤ *j* ≤ *N*. The quadrature weights can now be set to absorb the hyperbolic sampling, the power transform and the error model acting on the observed intensity and its associated standard deviation:

This thus yields a sum of weighted Rice functions that approximates the full likelihood function,

where *f*_·_(⋯) is the acentric or centric Rice function.

When the likelihood function is approximated using the power-transformed Laplace approximation instead of using the quadrature approach, we obtain a weighted Rice function

with the weight given by

where *E*_0_ = *x*_0_^γ^ and  is defined in expression (48).

## Appendix E Structure-factor amplitude estimation

In order to use an inflated variance modification as an approximation to the full numerical integration, we need to be able to estimate reflection amplitudes and their standard deviations from observed intensities and their standard deviations. While this process is normally performed using a standard French–Wilson estimation procedure, another route can be adopted following an approach developed by Sivia & David (1994 ▸). Assume a uniform, improper prior on *E*, such that

resulting in a conditional distribution

A normal approximation to this distribution can be obtained by the method of moments or, as performed here, by a maximum *a posteriori* approximation with a mean equal to the mode of the above distribution and a standard deviation estimated on the basis of the second derivative of the log-likelihood at the location of the mode:

An analytic expression is readily obtained, resulting in

The quality of this approximation will critically depend on the values of *Z*_o_ and σ_*Z*_^2^. Note that standard error propagation on *E*_o_ = *Z*_o_^1/2^ yields

which can be seen to be converge to expression (59) for the case where *Z*_o_ is significantly larger then σ_*Z*_.

## Appendix F Simulating synthetic data

Data for the numerical tests and benchmarks were obtained by sampling from the underlying distribution using the following procedure. For acentric reflections, ‘true’ complex structure factors and ‘perturbed’ complex structure factors are generated by successive draws from normal distributions with zero mean and specified variance:

where *N*(μ, σ^2^) denotes the normal distribution. For centric reflections, the following procedure is used:

Noise is added in the following fashion. Given a target variance σ^2^_target_, we generate ν + 1 normal random variates to compute the sample mean and sample variance:

Two error models are adopted in the described tests, namely a *fixed error ratio* or a *fixed error level*. In the fixed error ratio method, σ_target_ is different for every simulated intensity, and is chosen to be *Z*_True_/τ, where τ is equal to the desired  level. For the fixed error level method, σ_target_ is fixed at 1/τ for all intensities. A complete data set is simulated assuming a 9:1 ratio of acentric to centric reflections.
